# The Dynamics of Homologous Pairing during Mating Type Interconversion in Budding Yeast

**DOI:** 10.1371/journal.pgen.0020098

**Published:** 2006-06-23

**Authors:** Peter L Houston, James R Broach

**Affiliations:** Department of Molecular Biology, Princeton University, Princeton, New Jersey, United States of America; University of Milan–Bicocca, Italy

## Abstract

Cells repair most double-strand breaks (DSBs) that arise during replication or by environmental insults through homologous recombination, a high-fidelity process critical for maintenance of genomic integrity. However, neither the detailed mechanism of homologous recombination nor the specific roles of critical components of the recombination machinery—such as Bloom and Werner syndrome proteins—have been resolved. We have taken a novel approach to examining the mechanism of homologous recombination by tracking both a DSB and the template from which it is repaired during the repair process in individual yeast cells. The two loci were labeled with arrays of DNA binding sites and visualized in live cells expressing green fluorescent protein–DNA binding protein chimeras. Following induction of an endonuclease that introduces a DSB next to one of the marked loci, live cells were imaged repeatedly to determine the relative positions of the DSB and the template locus. We found a significant increase in persistent associations between donor and recipient loci following formation of the DSB, demonstrating DSB-induced pairing between donor and template. However, such associations were transient and occurred repeatedly in every cell, a result not predicted from previous studies on populations of cells. Moreover, these associations were absent in *sgs1* or *srs2* mutants, yeast homologs of the Bloom and Werner syndrome genes, but were enhanced in a *rad54* mutant, whose protein product promotes efficient strand exchange in vitro. Our results indicate that a DSB makes multiple and reversible contacts with a template during the repair process, suggesting that repair could involve interactions with multiple templates, potentially creating novel combinations of sequences at the repair site. Our results further suggest that both Sgs1 and Srs2 are required for efficient completion of recombination and that Rad54 may serve to dissociate such interactions. Finally, these results demonstrate that mechanistic insights into recombination not accessible from studies of populations of cells emerge from observations of individual cells.

## Synopsis

Genetic recombination not only promotes genetic diversity in a population but also insures the integrity of an organism's genome. Inappropriate or inefficient recombination drives tumor formation in cancers and underlies certain premature aging diseases in humans, such as Bloom's and Werner's syndromes. To gain insight into the role of recombination in these diseases, the researchers developed a means of observing recombination in individual cells. They accomplish this by repeatedly monitoring the positions of the two chromosomal segments participating in recombination. Their observations show that interacting strands undergo repeated association and dissociation prior to fully completing recombination. This view contradicts the accepted model of recombination as an uninterrupted, continuous process, but explains, as has been observed, how sequences from different regions of the genome might arrive at a single site after recombination. Moreover, their results show that mutants lacking the Bloom's or Werner's genes have difficulty bringing the interacting strands of DNA together. Such delays could lead to errors in genome structure that could account for the disease characteristics. In short, observing recombination in individual cells reveals unexpected but important features of this process.

## Introduction

Double-stranded DNA breaks (DSBs) occur spontaneously during replication and by exposure to certain genotoxic chemicals or ionizing radiation. Efficient repair of these DSBs can be accomplished non-conservatively by non-homologous end joining (NHEJ) or with exact fidelity using homologous recombination. Failure to repair these DSBs accurately can perturb the normal cell cycle progression and increase genomic instability, thereby promoting tumorigenesis.

The timing, choreography, and genetic dependencies of steps during DSB repair by homologous recombination in the budding yeast have been examined on populations of cells following synchronous initiation of a DSB [1–3]. Moreover, microscopic observation of live cells expressing specific green fluorescent protein (GFP)-tagged proteins has provided information on the temporal sequence and dependencies in recruitment of repair and recombination proteins to nuclear repair foci that form following initiation of DSBs [4]. These studies demonstrated that the repair of DSBs by homologous recombination occurs in several steps. First, shortly after formation of a DSB, signal transduction and nuclease modules recognize and process the DSB. The Mre11, Rad50, Xrs2 (MRX) module in conjunction with the Tel1 kinase tether the ends of the newly formed DSB and promote efficient nucleolytic processing of the break to generate 3′ single-stranded ends [4–6]. The single-strand binding complex, RPA, binds to the newly exposed single-stranded region. Rad51 then forms a nucleoprotein filament on the exposed single strands at the ends of the DSB. Rad52, Rad54, Rad55, and Rad57 also bind to the exposed single-strand region and promote formation and/or stabilization of the Rad51 nucleoprotein filament, in part by alleviating the inhibitory effect of RPA on Rad51 binding [2,3].

In the second step in DSB repair, the nucleoprotein filament performs a search for homology and associates with a donor sequence. Initial association likely occurs through a triple-strand association between the nucleoprotein filament with the duplex donor DNA, followed by isomerization to form a D-loop with the single-strand end of the DSB invading the duplex to pair with the template strand of the duplex [7]. Rad54 plays a role in strand invasion along with DNA polymerase holoenzyme [2], which extends the invading 3′ strand. In yeast, the joint between invading and template strands is resolved 2 to 4 h after DSB formation. However, most of these studies report only on the average behavior of populations of cells. Our results described in this report indicate that the discrete dynamics of the recombination process in individual cells are quite different.

A variety of helicases participate in recombination. One such helicase in *Saccharomyces,* Sgs1, is a member of the RecQ helicase family that resembles the WRN and BLM helicases defective in Werner and Bloom genome instability syndromes in humans [8]. Deletion of *SGS1* increases the frequency of gross chromosomal rearrangements and, in conjunction with deletion of a second related helicase gene, *SRS2,* causes a severe growth defect in yeast. This growth defect can be suppressed by mutations that inhibit initiation of homologous recombination, suggesting that Sgs1 and Srs2 prevent formation of aberrant, irreparable complexes that could arise during the process of homologous recombination [9]. Other studies have indicated that, despite the synthetic lethality of *sgs1* and *srs2,* the two corresponding proteins act at different steps in recombination, with Sgs1 helping to resolve recombination intermediates and Srs2 functioning to counteract initiation of recombination [10]. Moreover, these proteins appear to play critical roles during replication fork stalling, with Sgs1 promoting resolution of recombination-dependent structures that form at damaged replication forks and Srs2 assisting to prevent the formation of such structures [11]. Finally, while these studies suggest that Sgs1 and Srs2 suppress recombination, *sgs1* mutants are defective in DNA damage–induced heteroallelic recombination, suggesting that Sgs1 may promote recombination in certain contexts [9,12]. Consistent with this positive role of the yeast RecQ helicase in recombination, WRN protein is required to complete mitotic recombinants efficiently in unperturbed conditions in fibroblast cell lines [13]. Thus, we still do not have a clear conception of the function of RecQ helicases in recombination and a full appreciation of the mechanism underlying the pathological consequences of losing RecQ function in mammalian cells.

In order to address unresolved aspects of recombination, we have examined mating type switching in individual yeast cells. Mating type switching in yeast has been extensively used as a model system for homologous recombination [2,3,14,15]. Haploid *Saccharomyces* cells exist in one of two mating types, **a** or α, dependent solely on the particular allele present at the *MAT* locus on Chromosome III. Haploid cells can change mating type as often as every generation (reviewed in [16,17]) through recombination initiated by introduction of a DSB at the *MAT* locus, catalyzed by an endonuclease encoded by *HO* [18]. Switching then occurs by a gene conversion event, without associated crossover, that replaces the mating information at the *MAT* locus with the opposite mating information, **a** or α, present at either of two repository mating loci, *HML* and *HMR,* located at the opposite ends of Chromosome III. Mating type switching follows a precise developmental pattern that results from the intricate mode of transcriptional regulation of the *HO* gene and from a highly regulated interaction between donor and recipient loci [19–21]. Specifically, cell type dictates which donor locus is selected for participation in the gene conversion event. *MAT* **a** cells predominantly select *HML,* which normally contains α mating information, whereas *MAT*α cells select *HMR,* which normally contains **a** mating information. This selection process insures efficient mating type switching.

We have created strains for observing the interaction of *MAT* and its preferred donor locus during mating type switching in single cells in order to assess the discrete dynamics of the pairing process. The results of this analysis, described below, indicate that the dynamics of gene conversion during mating type interconversion are quite different, and much more rapid, in individual cells than would be predicted from previous studies on populations of cells. Furthermore, the dynamics of interaction of donor and recipient loci in various mutant backgrounds suggest unexpected roles for several components of the recombination machinery.

## Results

### Method for Observing Homologous Pairing in Live Yeast Cells

**Figure 1:**
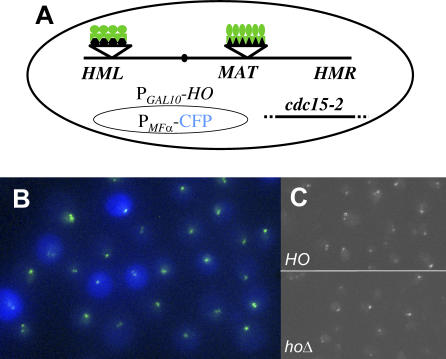
Visualization of Pairing between Donor and Recipient Loci during Mating Type Interconversion in Live Yeast Cells (A) Diagram of yeast cells used in this study, indicating the relevant structure of Chromosome III, which carries arrays of LacO sites at *MAT* and TetO sites at *HML*. The strain also constitutively expresses LacI-GFP and TetR-GFP protein fusions and contains a plasmid that carries a galactose-inducible *HO* gene and CFP under control of an α-cell type–specific promoter. (B) Fluorescence micrograph of a *MAT* **a** strain, Y3343, freshly transformed with plasmid B2609, 6 h after induction of *HO*. Green dots mark *HML* and *MAT* loci in each cell, and uniform blue staining indicates that the cell has undergone a mating type switch to *MAT*α. Cells are demarked by the diffuse cyan fluorescence, and the diffuse yellow fluorescence defines the nuclei. (C) Deconvolved and compressed images showing GFP foci in isogenic strains carrying or lacking P*_GAL1_*-*HO,* taken 90 min following induction with galactose.

We developed strains and a method to observe the pairing of homologous DNA loci during recombination in live yeast cells by adopting previously described methods to visualize discrete chromosomal loci and applying them to the process of mating type switching [22,23]. As diagrammed in Figure 1A, we inserted an array of *lac* repressor binding sites adjacent to a donor locus, *HML,* and an array of *tet* repressor sites adjacent to the recipient locus, *MAT,* and then expressed both GFP-lacI and GFP-tetR fusions in the strain [23,24]. We performed these experiments in a *cdc15–2* strain, which arrests at the non-permissive temperature just prior to exit from telophase. By shifting the strain from non-permissive to permissive temperature, cells synchronously enter G1 phase [25]. This insures that, at the beginning of our experiments, all cells have only one marked chromosome and are at the stage of the cell cycle in which switching normally occurs. Finally, the strain also carries the *HO* gene under control of the *GAL10* promoter to allow regulated induction of a DSB at the *MAT* locus.

To monitor cells under controlled conditions, we affixed yeast cells to the coverslip of a thermally regulated flow cell in which we could control both temperature and medium while repeatedly interrogating by fluorescence microscopy multiple fields of cells on the coverslip. Cells freshly transformed with the P*_GAL1_*-*HO* plasmid were pregrown in non-inducing medium at 23 °C and then shifted to 35 °C for 3 h. This arrests all the cells just prior to entry into G1 phase of the cell cycle. After attaching cells to the coverslip and centering the flow cell on the microscope, we initiated each experiment by simultaneously inducing the DSB at *MAT* and releasing cells from cell cycle arrest. This was accomplished by perfusing the flow cell with medium containing 2% galactose to induce HO and shifting the temperature of the flow cell to 23 °C. After DSBs were induced, we shut off *HO* expression to prevent subsequent rounds of recombination, by changing the medium flowing over the cells to one lacking galactose and containing 2% glucose. Since *HO* mRNA and HO protein have very short half-lives, no further DSB formation occurs much beyond this medium switch [26].

We confirmed that cells attached to the coverslip and treated as above switch efficiently and select the appropriate donor. We can determine by fluorescence microscopy at the single-cell level whether a cell has switched from *MAT* **a** to *MAT*α by including in the strain a P*_MF_*_α*1*_-CFP construct, which consists of the CFP coding region under control of the *MF*α*1* promoter. The *MF*α*1* promoter is active only in *MAT*α cells. Accordingly, a *MAT* **a** cell prior to switching does not express CFP, but after it switches to *MAT*α, it begins to accumulate CFP. We found that less than 1% of *MAT* **a** P*_MF_*_α*1*_-CFP cells without *HO* induction accumulated CFP at the end of the experiment, whereas approximately 50% of the cells that were induced for *HO* expression accumulated CFP (Figure 1B). The switching rate of 50% agrees well with that determined by genetic assay with the same strain induced for 1 h in liquid culture, indicating that manipulations in the flow cell and observations by fluorescence microscopy did not affect the process of switching. The rate is somewhat less than the 85% switching rate observed with wild-type *HO* cells and reflects a slight reduction in the initiation of switching under our induction conditions, rather than any alteration in donor preference (unpublished data). This agrees with Southern analysis and plate-based mating assays of our test strain, from which we concluded that under our induction conditions, approximately half of our test cells sustain a DSB at *MAT*.

### Pairing between Donor and Recipient Loci during Recombination Is Short and Reversible

**Figure 2:**
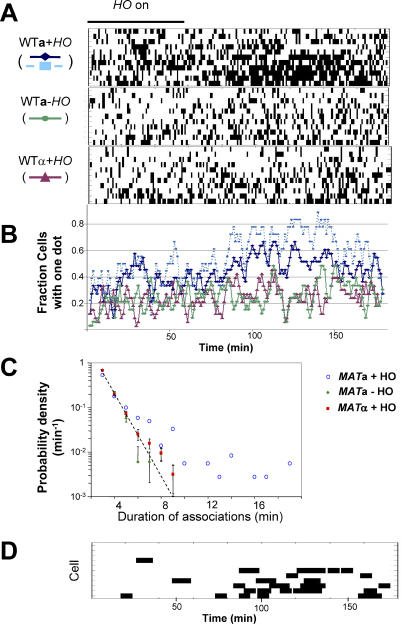
Pairing In Vivo between *MAT* and *HML* Increases following Introduction of a DSB at *MAT* (A) Shown are cell histories for 11 cells each from three strains, configured as diagrammed in Figure 1, indicating along the horizontal time line for each cell whether at each time point the cell presented a single dot (black bar) or two dots (white bar). Cells were interrogated at 1-min intervals for 3 h with galactose present (*HO* induced) for the first hour. Upper group: Y3343 (*MAT* **a**) transformed with B2609; middle group: Y3343 (*MAT* **a**) transformed with pRS415 [46]; and lower group: Y3342 (*MAT*α) transformed with B2608. (B) At each time point, the fraction of cells exhibiting a single dot was determined from the data underlying Figure 2A and plotted as a three-point running average versus time following addition of galactose. Legends are indicated to the left of the cell histories in (A). Data were calculated either including all 11 Y3343 (*MAT* **a**) cells transformed with B2609 (dark blue diamonds) or for only those six cells presumed to have sustained a DSB at MAT (light blue rectangles). The horizontal axis is aligned with the cell histories in (A). (C) The duration of periods of apparent continuous association between *MAT* and *HML*. The number of continuous, uninterrupted time points in which a cell shows only a single dot, were determined from the cell histories in Figure 2A. The number of periods of a given duration divided by the total number of periods (probability density) is plotted versus the duration of apparent association for each strain shown in 2A. *MAT* **a** + *HO* (light blue circles); *MAT* **a** − *HO* (green diamonds); and *MAT*α + *HO* (red squares). Error bars are estimated by dividing the data successively into random halves and computing the standard deviation among 20 random halves. The dashed line shows a fit of the combined values for *MAT* **a** − *HO* and *MAT*α + *HO* cells to a normalized, exponential distribution. Under this assumption, the *p*-value for the likelihood that the data for *MAT* **a** + *HO* would arise by chance is less than 10^−5^. (D) Data from Figure 2A for *MAT* **a** + *HO* cells is replotted to show only those pairing events of duration longer than 6 min.

To monitor pairing of *MAT* and *HML* following initiation of a DSB at *MAT,* we treated *MAT* **a** cells as described above and then interrogated multiple cells every minute for 3 h. We determined in each cell, at each time point, whether the nucleus contained two GFP dots or a single GFP dot. In the former case, we assume that the *MAT* and *HML* loci are unpaired. In the latter case, we assume that the two loci are either paired or simply too close to be resolved. Representative images from experiments in which *HO* was induced or not induced are shown in Figure 1C, demonstrating the resolution of the two loci in the test strains and indicating that all cells can be readily scored. In Figure 2A, we represent the histories of association of *MAT* and *HML* as determined by this method following *HO* induction for 11 wild-type *MAT* **a** cells, which normally use *HML* as the preferred donor. In addition, we examined 11 wild-type *MAT*α cells following *HO* induction. *MAT*α cells normally use the untagged *HMR* locus as donor and thus *MAT* would not be expected to pair with *HML* in this strain. Finally, we examined 11 wild-type *MAT* **a** cells in the absence of *HO* induction, in which no recombination should occur. In these representations, each line describes the history of a single cell; a white bar represents the presence of two dots in the cell's nucleus at that time point and a black bar represents the appearance of only a single dot.

These interrogations clearly show that, relative to the behavior of *MAT* **a** cells in which *HO* was not induced, *MAT* **a** cells in which *HO* was induced exhibited a statistically significant increase in the overall proportion of time *MAT* and *HML* were in close proximity. Moreover, cells from the induced culture divided into two discrete populations. In one group, cells exhibited a pattern of association that was indistinguishable from those of uninduced cells, whereas in the other group, cells exhibited a succession of extended associations between *MAT* and *HML* lasting from 7 to 15 min, starting approximately 80 min after initiation of *HO* expression. The fraction of cells exhibiting the extended association matched the fraction of cells that ultimately undergo gene conversion, and we believe that cells showing extended association were those in which cleavage at *MAT* had occurred.

A distinction between the association patterns in induced and uninduced cells is also evident by calculating the fraction of cells containing a single dot at each time point. As shown in Figure 2B, cells in which *HO* was not induced show a uniform, low level of association between *HML* and *MAT* throughout the experiment. On the other hand, *MAT* **a** cells in which *HO* was induced exhibit increased association of *HML* and *MAT* starting from 80 min after initiation of induction of *HO* and lasting for approximately 1 1/2 h.

We also noted that, despite the fact that more than half of the cells expressing *HO* undergo gene conversion of *MAT* using *HML* as donor, continuous pairing between *MAT* and *HML* occurs for no longer than 17 min in any cell. We evaluated the number and duration of events in which *HML* and *MAT* appear to be in continuous association, i.e., cases in which a cell presents a single dot in an uninterrupted series of time points. As shown in Figure 2C, in uninduced *MAT* **a** cells or induced *MAT*α cells, the log of the number of association events of a given duration plotted versus the duration of association exhibits essentially a linear relation, consistent with a stochastic process in which the likelihood that *HML* and *MAT* are in close proximity at any time is independent of whether they were in close association at the previous or subsequent time point. Only one event longer than 6 min was observed in uninduced cells. In contrast, induced *MAT* **a** cells exhibit a statistically significant excess of events of duration longer than 6 min. These extended associations occur for an average of 10 min, predominantly between 80 and 160 min postinduction (Figure 2D). Unexpectedly, each cell undergoes more than one prolonged association event. Moreover, during the period between two extended associations, the average distance between the two spots (0.49 ± 0.25 μm) is equivalent to that in uninduced cells (0.51 ± 0.24 μm), which do not undergo switching. Thus, we conclude that the multiple associations of donor and recipient in a single cell are independent events, suggesting that the pairing of donor and recipient loci is readily reversible during recombination.

Finally, we note from the data in Figure 2 that, in contrast to the pattern seen with *MAT* **a** cells, *MAT*α cells, which do not use *HML* as the preferred donor, do not exhibit an enhanced association between *MAT* and *HML* over the course of the experiment. Rather, induced *MAT*α cells present a pattern indistinguishable from that obtained in uninduced cells. This suggests that the cleaved *MAT* locus does not spend significant time in association with the non-preferred donor locus during mating type switching.

### Sgs1 and Srs2 Are Required for Stable Pairing between Donor and Recipient Loci during Switching

**Figure 3:**
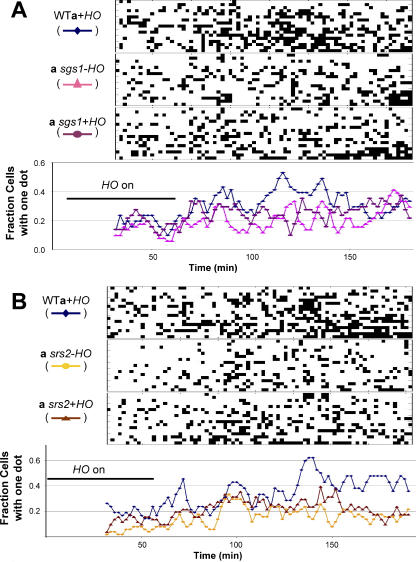
*HML* and *MAT* Fail to Pair in *sgs1* or *srs2* Mutants Data were obtained for 17 cells under each condition and presented as described in Figure 2 for *sgs1* (A) and *srs2* (B) mutant strains, with the difference that cells were interrogated every 2 min starting 30 min after addition of galactose.

We anticipate that assembly and resolution of recombination intermediates during switching would require the activity of DNA helicases, and several genes encoding helicases have been implicated in recombination and repair. In an attempt to clarify the role of various helicases in recombination, we examined the extent of association between *MAT* and *HML* in strains deleted for individual helicase genes. As shown in Figure 3, the level of pairing between *HML* and *MAT* in a *MAT* **a** *sgs1*Δ (Figure 3A) or a *MAT* **a** *srs2*Δ (Figure 3B) mutant following *HO* induction was significantly reduced compared to that observed in wild-type cells. The level of pairing in the induced *srs2*Δ strain was slightly higher than that in the same strain lacking *HO* but was certainly significantly lower than that observed in the wild-type strain. More dramatically, the level of apparent pairing in the induced *sgs1*Δ strain was not significantly different than that in the same strain lacking *HO*. Thus, association of the DSB with the donor locus appears to be delayed in both mutants. These results would predict that *sgs1* or *srs2* mutants should be defective in mating type switching.

To confirm the roles of Sgs1 and Srs2 in repair of DSBs during mating type switching, we examined the effect of introducing these mutants into wild-type *HO* strains. A diploid *HO/HO* strain, which we used previously to determine switching frequencies under normal conditions [27], was rendered heterozygous for deletion of *SGS1* or *SRS2*. After sporulation and dissection, we examined the behavior of cells and the switching pattern during outgrowth of spore clones. As seen in Table 1, *HO SGS1* and *HO SRS2* clones showed normal growth and switching patterns. Similarly, *ho sgs1* and *ho srs2* control strains grew indistinguishably from *ho SGS1* and *ho SRS2* strains (unpublished data and [9]). In contrast, *HO srs2* spore clones were smaller than their sister *HO SRS2* spore clones, and pedigree analysis of cells following germination indicated that mother cells in the lineage exhibited delayed cell cycle progression or failed to divide at all. This growth pattern is similar to, but less severe than, the pedigree of death that occurs in *HO* yeast strains incapable of completing HO-induced switching [28], which we observe with *rad52 HO* and *rad54 HO* spores (Table 1). Spore clones carrying an *sgs1* deletion did not show as extensive delays in growth as did *srs2* spore clones, but the viability of mother cells in pedigrees was clearly less than that seen with sister *SGS1* spore clones (unpublished data). These results are consistent with the assumption that *srs2* mutants, and to a lesser extent, *sgs1* mutants, fail to efficiently heal DSB generated in lineages of *HO* haploid cells.

**Table 1:**
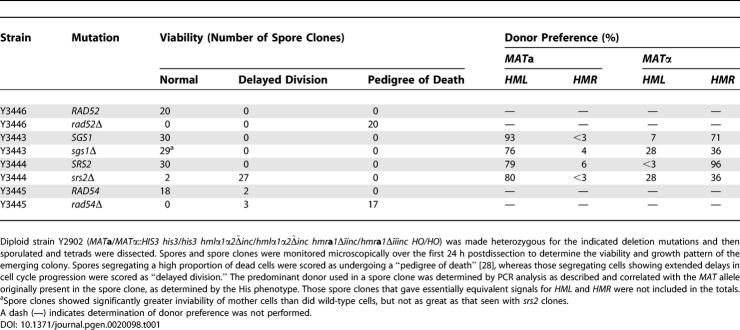
Switching Efficiency in Mutant Strains

We also find that, although both *srs2* and *sgs1* mutants exhibited normal donor selection in a *MAT* **a** background, both mutants showed essentially random selection of donor loci following switching in a *MAT*α background (Table 1). This observation confirms that the mutants exhibit a delay in repairing the DSB during mating type switching. In cells in which repair of the *HO*-induced DSB at *MAT* is delayed, resection of the DSB into the adjacent coding region results in deletion of the *MAT* locus, rendering the cell temporarily *mat*-null. Since *mat*-null cells exhibit a *MAT* **a** phenotype, donor selection in *MAT* **a** cells would be unaffected by this delay-induced resection. However, the delay-induced resection in a *MAT*α would render the cell phenotypically *MAT* **a**, changing the donor preference from *HMR* to *HML*. Thus, the extent to which *MAT*α cells select *HML* as donor reflects the extent of delay in the repair of the DSB. Accordingly, our results confirm that Srs2 and Sgs1 are both required for timely repair of DSBs.

### Rad54 Is Not Required for Stable Pairing between Donor and Recipient Loci during Switching

*RAD54* encodes a member of the Swi2/Snf2 family of DNA-stimulated ATPases, and loss of *RAD54* function substantially diminishes homologous recombination and renders cells sensitive to ionizing radiation. Consistent with previous reports that Rad54 is required for completion of DSB repair [29], *HO rad54* spore clones yield microcolonies in our donor preference assay, resulting from extensive death of mother cells during growth of the clone (Table 1). Previous studies on the precise role Rad54 plays in recombination have been inconsistent, with some evidence pointing to its involvement in initial formation of the Rad51 presynaptic filament and other evidence suggesting that it functions after formation of the presynaptic complex [2,3]. To help resolve this issue, we examined pairing of *MAT* and *HML* following *HO* induction in a *rad54*Δ mutant.

**Figure 4:**
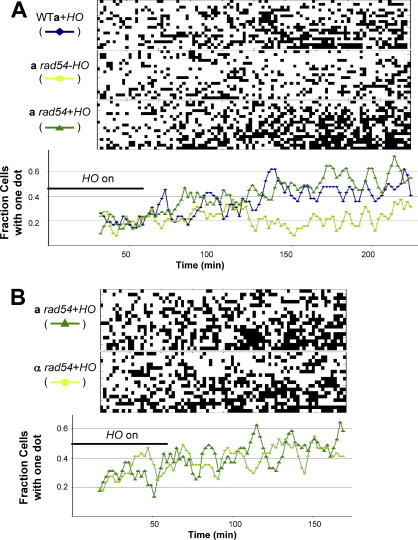
Rad54 Is Not Required for DSB-Induced Pairing of *MAT* and *HML* Data were obtained and presented as described in Figure 2 for *MAT* **a** *rad54* cells (A) and for *MAT*α *rad54* cells (B).

As evident in Figure 4A, the two loci paired as efficiently in the *rad54* mutant as in wild-type cells. Surprisingly, *MAT* and *HML* also paired efficiently in a *MAT*α *rad54* mutant strain (Figure 4B), despite *HML* not being the preferred donor for switching in *MAT*α cells. This result is quite distinct from that observed with *HO*-induced *RAD54 MAT*α cells, which, as shown in Figure 2, show no prolonged association between *MAT* and *HML*. Therefore, association between donor and recipient loci proceeds efficiently in the absence of Rad54 function and, furthermore, association with the inappropriate donor occurs as frequently as does association with the preferred donor.

## Discussion

To study the dynamics of recombination, we have developed a real-time assay for homologous pairing in individual living cells. The results from this assay correlate well with previous observations on the time course of the recombination reaction obtained with populations of cells, but also reveal unanticipated aspects of recombination not previously accessible through population studies. The system we examined—mating type interconversion following synchronous cleavage of the *MAT* locus by *HO* induction in *Saccharomyces*—has been used extensively as a model system for homologous recombination [2,3,14,15]. Previous studies examining products and intermediates of recombination in vivo by PCR or Southern analysis indicate that strand invasion begins approximately 90 min following *HO* induction and ends approximately 90 min later [2,14]. Furthermore, by using chromatin immunoprecipitation to quantify in vivo association of recombination proteins with regions of the genome, several studies determined that Rad51 initially associates with the DSB at *MAT* approximately 30 min following *HO* induction—coincident with DSB formation—and with the donor locus, *HML,* approximately 30 min later [2,3]. This association continues for up to 3 h. These results suggest that synapsis between the DSB and the donor locus begins approximately 60 min following *HO* induction and that the two loci remain physically associated for up to several hours. Our data are consistent with these population studies in that we observe an overall increase in the average association of *MAT* and *HML* beginning 80–90 min following *HO* induction and lasting for 60–90 min.

**Figure 5:**
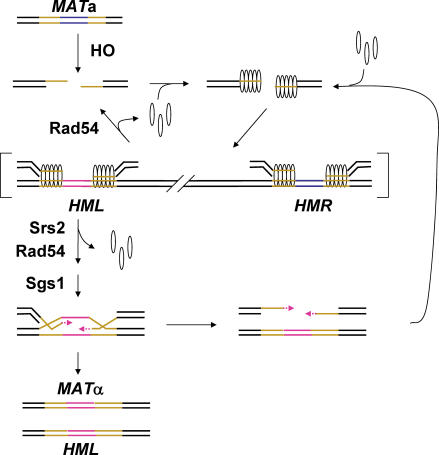
Model for DSB-Induced Pairing of *MAT* with Donor Loci Following HO-induced double-strand cleavage of *MAT,* Rad51 (ellipses) binds to the resected single-strand ends of the DSB and promotes their association with both *HML* and *HMR* via homology present at all three loci. Blue- and pink-colored regions at the mating type loci represent allele-specific DNA sequences, which are bracketed by sequences in common among all three loci (shown in brown). Dissociation of these initial complexes, suggested to be paranemic joints in this illustration, is stimulated by Rad54-promoted removal of Rad51. Since these associations are seen only in *rad54* cells, we assume that they are too short-lived to be evident in wild-type cells, and thus are indicated in brackets. We propose that Rad54 also stimulates conversion of the initial joint complex into an initial D-loop structure, also by promoting removal of Rad51, allowing subsequent extension of the heteroduplex between the invading strand and donor locus, and elongation of the invading strand. These subsequent steps are promoted or stabilized by Srs2 and Sgs1. Our results suggest that one or more of the later-stage intermediates in recombination, perhaps even after partial extension of the invading strand, are also reversible prior to completion of the gene conversion event. The dissociated, but incompletely healed, *MAT* locus then would recycle through Rad51 filament formation and reassociation with the donor locus, ultimately yielding a completely reconstituted *MAT* locus.

Monitoring the association of *HML* and *MAT* over time in individual cells revealed aspects of synapsis not evident from these population studies. First, we observed a statistically significant increase in the number of periods of extended association of *MAT* and *HML* in induced versus uninduced cells, although the durations of these associations were in general no longer than 17 min and averaged approximately 10 min. Thus, distinct periods of synapsis in individual cells are much shorter than would be expected from population studies. Second, individual cells exhibit several distinct periods of extended association following *HO* induction. Since *HO* protein is not present at these later times and since the same pattern occurs in *rad54* mutants unable to complete recombination, these sequential associations do not represent sequential rounds of resolution and re-initiation of recombination. Also, these multiple associations are unlikely to be a single synaptic junction punctuated by dynamic stretching, since the distributions of distances between the two loci in the interval between two such extended associations are identical to those in uninduced cells. Rather, we would interpret these observations to suggest that the Rad51-mediated complex formed between the DSB and the template is reversible. For example, the initial Rad51-mediated triple-strand complex consisting of the Rad51-coated single-strand DNA bound to the homologous double-strand region of the donor template could be resolved in either two ways: isomerization to yield a single-strand invasion creating a D-loop on the template, or disassociation to return to the pre-complex state. Alternatively, D-loop formation could be the reversible step, either before or after extension of the single-strand primer. Our results suggest that either or both of these initial interactions between the DSB and the template can occur several times before completion of recombination. Such cycles for strand invasion have been suggested to occur during repair of DSBs in *Drosophila* [30]. The results from those studies with *Drosophila* would suggest that dissociation could occur not only after D-loop formation but also after partial extension of the primer, as depicted in Figure 5. Finally, we note that the multiple, stochastic associations between the DSB and the donor locus in individual cells account for the apparent extended association between the two loci as measured on a population basis.

We explored some of the genetic requirements for homologous pairing in our system. We first examined the role of the helicases encoded by *SGS1* and *SRS2* and found that inactivation of either of these genes delayed sustained association between *MAT* and *HML*. We confirmed this result obtained from the physical pairing assay by showing that repair of DSBs during mating type switching is delayed in *HO* spore clones carrying *sgs1* or *srs2* mutations. Thus, each of these genes appears to be independently required for efficient homologous pairing in our system, an observation consistent with previous studies suggesting that, despite their synthetic lethality, Sgs1 and Srs2 play independent roles in recombination [9,12].

Our results are somewhat unexpected since previous genetic and biochemical studies have implicated Srs2 in inhibiting initiation of recombination by promoting disassembly of Rad51 filament complexes [31–33], whereas Sgs1 and other RecQ helicases are likely involved in resolution of recombination intermediates [10,34–38]. Thus, we might have anticipated that *sgs1* or *srs2* mutants would exhibit prolonged association of *MAT* and *HML* in our assay rather than reduced association. However, previous studies have shown that, although *sgs1* mutants exhibit a hyperrecombination phenotype under some conditions, they are hyporecombinogenic for DNA damage–induced heteroallelic recombination [9,12], a situation similar to DSB-induced mating type switching. In addition, Sgs1 is required for telomere elongation in the absence of telomerase, apparently through recombination via a break-induced repair process that is mechanistically similar to the DSB-induced gene conversion we have studied here [39]. Thus, Sgs1 could be required to stabilize the initial joint formed between a DSB and the donor by promoting formation of an extended D-loop or initiation of synthesis to extend the invading 3′ end. In a similar vein, the effect of inactivation of *SRS2* on recombination depends on the particular assay used. Perhaps most relevant to our observations, Aylon et al. [40] examined gene conversion of a *ura3* allele from an ectopic site of homology following introduction of an *HO*-induced DSB within the *ura3* allele, an assay quite similar to mating type switching. They found that inactivation of *SRS2* substantially reduced the level of strand invasion and subsequent extension of the invading strand and concluded that only a small subpopulation of *srs2* cells are able to complete recombination, whereas the majority of cells behaved as if no homology to the DSB were present in the cell. This is consistent with results from Pâques and Haber suggesting that Srs2 functions to extend and stabilize initial joints between the ssDNA-Rad51 nucleofilament and the donor locus [41]. Thus, both Sgs1 and Srs2 have been implicated independently in stabilizing initial joint formation following DSB-induced recombination, consistent with our current observations.

We also used our assay to examine the role of *RAD54* in pairing in vivo. Rad54, a dsDNA-dependent ATPase of the Swi2/Snf2 family of chromatin remodeling enzymes, is required for resistance to ionizing radiation and repair of DSBs by homologous recombination [7,29]. Rad54 interacts with Rad51 in vivo, and addition of Rad54 to Rad51-mediated recombination reactions dramatically stimulates homologous pairing in vitro. In addition, Rad54 is essential for DNA strand invasion reactions using chromatin-packaged substrates [42]. Two studies recently used chromatin immunoprecipitation to examine the in vivo requirement of Rad54 for association of Rad51 with *MAT* and *HML* following initiation of a DSB at *MAT*. Wolner et al. [3] found that Rad51 association with either locus was essentially eliminated in a *rad54*Δ mutant background and provided evidence that Rad54 promotes formation of the Rad51 presynaptic filament. On the other hand, Sugawara et al. [2] found that Rad51 could, in fact, associate with *MAT* and *HML* in the absence of Rad54, but the synaptic complex could not proceed to a mature strand invasion complex in the absence of Rad54.

We find that *MAT* and *HML* associate as well in a *rad54* mutant as they do in a wild-type strain. Moreover, we find that *MAT* associates frequently with *HML* even in a *MAT*α strain, in which *HML* is the non-preferred donor. One possible explanation for these associations between *MAT*α and *HML* is phenotypic switching of mating due to delay in resolution of the *MAT* DSB, as we proposed for *sgs1* and *srs2* mutants. However, since the timing of the observed associations in *rad54* mutants is the same as that seen in wild-type cells, in which no phenotypic switching occurs, we conclude that the inappropriate associations are not the result of phenotypic change in mating type. Rather, our results suggest that Rad54 plays a critical role in recombination at a step beyond initial synapsis. This conclusion is consistent with the observations above from Sugawara et al. [2] and also with subsequent work by Wolner and Peterson [43]. Although Rad54 may also have a role in vivo in promoting Rad51 nucleofilament formation, our results would suggest that this is not the rate-limiting step in recombination stimulated by Rad54. Our results can be explained by the proposal, based on the observation that Rad54 stimulates dissociation of Rad51 from dsDNA, that Rad54-stimulated removal of Rad51 promotes either dissociation of the paired loci or conversion of the initial synaptic intermediate into a D-loop and the subsequent extension of the heteroduplex region [44]. Thus, in the absence of Rad54, pairing between the DSB and either donor locus should still occur, but the formation of a D-loop and subsequent elongation of the invading strand would not. This model would account for the fact that we observe pairing of *MAT* and *HML* in the non-preferred mating background, reflecting formation of Rad51-mediated pairing of MAT with either donor locus via the common homology at all three loci. We would propose that these initial pairing events would be eliminated in a Rad54-dependent fashion, either reversing the initial pairing or promoting progression in the appropriate partner to productive recombination.

A model for recombination during mating type switching is presented in Figure 5. Our results suggest that following HO-induced cleavage of *MAT,* the locus associates readily and reversibly with both *HML* and *HMR*. Donor selection would then occur at a subsequent step, for instance in the isomerization from the initial synaptic intermediate to the D-loop, upon recruitment of DNA polymerase or with extension of the invading strand. Cell type–specific loading of factors promoting D-loop formation and strand extension at the preferred donor locus would then fix donor preference. Our results also suggest that this step is stimulated by both Sgs1 and Srs2. Our results further indicate that, even in the presence of Rad54, the association between donor and template are reversible. This would suggest that even after D-loop formation, the donor and recipient loci can dissociate. Moreover, given recent results suggesting that a double-strand gap requires multiple cycles of strand invasion, synthesis, and dissociation of the nascent strand during repair of a DSB in *Drosophila* [30], this reversibility could occur after partial extension of the invading strand, as represented in Figure 5. Further analysis should resolve precisely at what steps recombination is reversible. However, these results clearly indicate that the recombination process is reversible at a much later stage than previously anticipated and raises a novel mechanism for generating genetic diversity.

## Materials and Methods

### Plasmids and strain construction.

Plasmid pPJS218 (HIS3 TetR-GFP LacI-GFP) has been previously described [45]. Plasmid B2609 (*LEU2 CEN4 ARS1* P*_GAL1_*-*HO* P*_MF_*_α*1*_-CFP) was constructed by in vivo recombination by co-transforming a *leu2 trp1* yeast strain with plasmid B2686 *(LEU2 TRP1 CEN4 ARS1* P*_GAL1_*-*HO)* and two PCR fragments, one spanning the *MF*α*1* promoter and one spanning the coding sequence of CFP from plasmid pDH3 (http://depts.washington.edu/~yeastrc). Both PCR fragments carried 50 base pair ends with homology to direct recombination with the appropriate adjacent fragments. Leu^+^ FAA-resistant transformants were selected and plasmid was recovered by transformation into Escherichia coli. Plasmid B2608 (*LEU2 CEN4 ARS1* P*_GAL1_*-*HO* P*_MF_* **_a_** *_1_*-CFP) was constructed in an analogous fashion using the *MF* **a** *1* promoter. Plasmid structures were confirmed by functional assays and restriction analysis.

Strains used in this study are listed in Table 2 and were all obtained from the S288C-derived haploid strain S150-2B. Construction of strains carrying DNA binding site arrays adjacent to *MAT* and *HML* have been described previously [45]. GFP fusions were introduced by integrative transformation of plasmid pPJS218, selecting His^+^ transformants. The *cdc15–2* allele was introduced into S150-2B by first integrating plasmid B2400 (pRS406-*cdc15–2*) into the CDC15 locus and then selecting FOA-resistant revertants of selected transformants. FOA-resistant, temperature-sensitive isolates were scored microscopically for terminal arrest phenotype to confirm the presence of the *cdc15–2* allele. The *cdc15–2* allele was then introduced into the tagged strains by genetic crosses. Deletion alleles were introduced as needed by selecting transformants resistant to G418 (200 μg/ml, CalBiochem, San Diego, California, United States) following transformation with PCR products using DNA from the appropriate strain from the *kanMX* deletion collection, and primers 500 base pairs upstream, 5′, and downstream, 3′, of the gene.

**Table 2:**
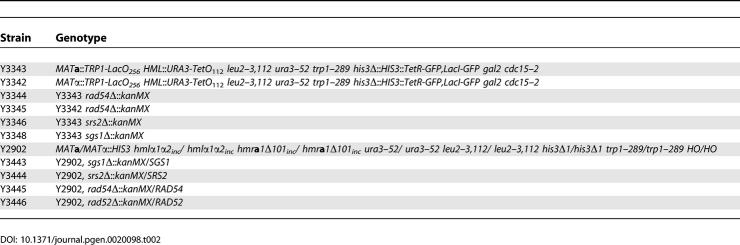
Strain List

Strains for donor preference were obtained by transformation of strain Y2902 using PCR products from the deletion collection as described above. Donor preference in spore clones of mutant strains were determined as described previously [27].

### Microscopy methods.

The yeast strains were freshly transformed with plasmid B2609 or B2608 and grown 3 d at 23 °C on SC-leu media. Three transformants of each strain were inoculated to 20-ml SC-leu broth + 2% glucose and grown overnight at 23 °C. Cultures were then diluted into SC-leu broth + 2% raffinose and lacking glucose to a density of 10^6^ cells/ml and incubated for 3 h at 37 °C to arrest growth and render *GAL1-HO* readily inducible. Cells from each culture were examined by fluorescence microscopy to confirm that most exhibited two nuclear GFP dots and none expressed CFP. Cells from cultures with these properties were applied to a microscope coverslip (No.1 22×60 mm, Corning, Corning, New York, United States) precoated with Concanavalin A (Sigma, St. Louis, Missouri, United States) as described (http://www.cgr.harvard.edu/thornlab/protocols/ConA.htm). Samples of test and control cultures were applied to the same coverslip but separated by a hydrophobic Pap pen (Daido Sangyo Co. Ltd., Tokyo, Japan) to preclude mixing. The cells were mounted on a flow cell (BioSurface Technologies, Bozeman, Montana, United States) and perfused with SC-leu medium + 2% raffinose as the sole carbon source, maintained at 37 °C with a thermocouple detector and a feedback-modulated heated stage. Cells from several fields of each strain were repeatedly interrogated every 1 or 2 min using a Nikon Eclipse TE200 microscope with 100× objective (1.4 aperture) and a planapochromatic light source (Nikon, Tokyo, Japan). Approximately 5 min after mounting the coverslip on the flow cell, the temperature was shifted to 23 °C to release the *cdc15–2*–imposed cell cycle arrest, and medium perfusing the flow cell was changed to SC-leu + 2% galactose. This time was set as the zero time point, and galactose perfusion was maintained for 60 min, at which time medium was switched to SC-leu + 2% glucose. At each interrogation, we captured 30 0.4-μm Z-sections of a 512 × 512 pixel image (0.1 μm per pixel), and prior to analysis, the images were processed by five cycles of deconvolution using Deltavision SoftWoRx v. 2.5 (Applied Precision, Issaquah, Washington, United States). The locations of GFP dots in each cell in a field were determined from the identification of the maximum intensity in the three-dimensional images. Distance between the separable dots was determined by applying the Pythagorean triangulation to the Cartesian coordinates of the maxima using Softworx pick points function and processed with the timedist and yeastparser programs (John Houston, Multimedia Gaming, Austin, Texas, United States). Those cells in which only a single intensity maximum was observed were defined as having a single dot, i.e., the two loci were closer than could be resolved by our microscopy.

## Abbreviations

DSB: double-strand DNA break
GFP: green fluorescent protein
